# The Impact of Transformational Leadership on Physicians’ Performance in China: A Cross-Level Mediation Model

**DOI:** 10.3389/fpsyg.2021.586475

**Published:** 2021-03-09

**Authors:** Haiyun Chu, Binbin Qiang, Jiawei Zhou, Xiaohui Qiu, Xiuxian Yang, Zhengxue Qiao, Xuejia Song, Erying Zhao, Depin Cao, Yanjie Yang

**Affiliations:** Harbin Medical University, Harbin, China

**Keywords:** transformational leadership, physicians’ performance, achievement motivations, coping styles, health care

## Abstract

Transformational leadership has been becoming increasingly vital to the provision of high-quality health care, particularly during major public health emergencies. The present study aims to investigate the impact of transformational leadership on physicians’ performance and explore the cross-level underlying mechanisms with achievement motivations and coping styles among Chinese physicians. During 2017–2019, 1,527 physicians of 101 departments were recruited from six hospitals in China with a cluster random sampling method. Participants completed several questionnaires regarding their job performance, achievement motivations, coping styles, and transformational leadership. Multilevel mediation effects were tested using cross-level path analysis. The result of this study indicated that transformational leadership was applied well in Chinese medical settings with a score of 101.56 ± 6.42. The hierarchical linear model showed that transformational leadership had a cross-level direct positive effect on physicians’ performance (β = 1.524, *p* < 0.05). Furthermore, results of cross-level path analyses revealed that transformational leadership contributed to physicians’ performance by sequentially influencing achievement motivations first and then coping styles. In addition, the path “transformational leadership → positive coping (PC) style → physicians’ performance” showed the strongest cross-level indirect effect. In summary, public health leaders should enhance physicians’ performance by promoting individual development, especially achievement motivation and PC style.

## Introduction

With a large 1.4 billion population and an ongoing social transition, China currently faces an ever-growing number of public health challenges, including infectious diseases, chronic diseases, obesity, alcohol and drug disorders, climate change, food insecurity, unbalanced demand, and supply of health services, etc. (The Lancet and Public Health, 2018). In particular, outbreaks of major public health emergencies, such as the Ebola, SARS, and COVID-19 crises, have been becoming the most challenging public health threat and requiring higher demands on health systems across the world. Prevention and control of major epidemics are dynamic to adapt to meet the needs of evolving public health practice. In hospitals, the emergency changes require medical leaders to lead effectively toward the new setting.

Transformational leadership, recognized as a contributor to greater outcomes of employee and organization in transformational development, has become increasingly vital to the provision of high-quality medical service (Masood and Afsar, 2017). The characteristic feature of transformational leadership is idealized influence, inspirational motivation, intellectual stimulation, and empowerment (Shaw et al., 2018). In medical settings, transformational leaders generally inspire public health staff to share the sacred mission, stimulate their intelligence, and provide individualized consideration (Fischer, 2016; Pearson, 2020). When leaders used more transformational leadership, employees (especially those who had a high need for leadership) were more likely to engage in work and better performance (Breevaart et al., 2016). In Chinese health care settings, the physicians’ salaries are relatively low (Zhang and Liu, 2018); however, it is an interesting phenomenon that when immediate leaders embody the principles of transformational leadership, physicians perform better and more productively. Because of the largest population and the imbalance of health care development in China, Chinese physicians face the largest health care population and heavy work worldwide. They not only serve patients but also support specific groups who need health care services, such as the aged, the pregnant, children, etc. Under overwhelming pressure, physicians are physically and mentally exhausted (Qiao et al., 2016), leading to low work efficiency, low quality of medical service, and low job performance. As a crucial team-level characteristic of the health care system, how does transformational leadership’s power make physicians’ performance better? Unfortunately, little is known about the underlying mechanisms. To address this issue and supplement previous works, the present study combined team-level and individual-level characteristics in health care settings to explore the cross-level underlying mechanisms of the positive impact of transformational leadership on physicians’ performance.

According to Transformational Leadership Theory, a transformational leader attaches importance to promoting followers’ intrinsic motivation from low-level needs to high-level needs (Lo et al., 2018). Public health leadership can support the professional development of the public health workforce. Leaders with transformational leadership have a profound impact on employees by changing their cognition and behavior patterns (Lord et al., 2017). Hence, we reasonably speculate that achievement motivations and coping styles may play important roles in the relationship between transformational leadership and job performance.

Achievement motivations are defined as the internal drivers of individuals to pursue valuable goals, achieve high performance, and strive for success (Elliot et al., 2018). McClelland believed that different people had different achievement motivations; individuals with a high level of achievement motivation tended to take responsibility and risk, enjoy their work, work hard, and keep forging ahead actively (McClelland et al., 1973). There are two psychological tendencies in competition: the motivation to pursue achievement (MS) and the motivation to avoid failure (MF) (Michou et al., 2014). The former is generally positively correlated with outstanding achievement, while the latter is always related to fewer errors. Since the last century, a number of researches have already been carried out to explore the link between work-related motivations and job performance (Cerasoli et al., 2014) and demonstrated that achievement motivations benefited work life and work outcomes (Suárez-Álvarez et al., 2013). Individuals with higher MS perform better and get high work efficiency. Meanwhile, health worker motivations would be influenced by leadership in medical settings (Musinguzi et al., 2018). Transformational leadership serving a higher purpose motivates public health staff to plunge into their work (Ree and Wiig, 2019), and their motivations change their behavior styles and productivity (Judson et al., 2015).

Coping styles refer to the strategies or means that people adopt to cope with internal–external demands and conflicts (Houslay et al., 2018). Positive coping (PC) and negative coping (NC) are two common opposite coping styles. In a work setting, individuals with PC more often assess risks and difficulties objectively, positively deal with work challenges, and ultimately solve problems. With positive thinking and optimistic psychology, most of these people perform excellently in their work. On the contrary, employees with NC are more inclined to step away from challenges and avoid risks in their work. Most of them generally end with undesired outcomes and mediocre performance. Heavy clinical workloads, high work pressure, and the nature of life-threatening tasks lead to differences in health workers’ performance (Udod et al., 2017). A study in health care settings revealed that PC strategies buffered the negative effects of work stress on job performance while NC strategies increased the negative effects (Li et al., 2017). In Chinese medical settings, it is an interesting topic of how different coping styles affect physicians’ performance under transformational leadership.

Despite growing evidence for the positive impact of transformational leadership in public health settings, the underlying mechanisms remain poorly defined. As a result, managers only highlight the importance of transformational leadership; however, a more specific and more effective way to play a leadership role is still undiscovered. Overlooking possible processes of cognitive and behavioral changes of physicians limits the practical implications of research and leaves the question of causality unaddressed. To better understand the application of transformational leadership in Chinese hospital settings and explain the cross-level underlying mechanisms of the relationship between transformational leadership and physicians’ performance, this study sought to examine two contents: ➀ how transformational leadership directly impacted physicians’ performance and ➁ how transformational leadership affected individual development of physicians, such as achievement motivations and coping styles that could potentially improve physicians’ performance.

## Materials and Methods

### Aims, Study Design, Participants, and Procedures

This study aims to investigate the application of transformational leadership in Chinese hospitals and discover the cross-level underlying mechanisms of the impact of transformational leadership on physicians’ performance.

The present study was cross-level research conducted in Harbin, Heilongjiang Province, China, during 2017–2019. We recruited physicians with a cluster random sampling method from six hospitals. First, six hospitals were selected from all hospitals randomly; next, we calculated the distribution of physicians from these hospitals as the proportion of participants; then, we stratified the physicians into different departments according to the employment contract; finally, we selected departments randomly, and all physicians in these departments were recruited as participants of this study.

Eventually, we received a total of 1,527 physician responses from 101 departments of six hospitals. The average age of the participants was 36.55 ± 7.96 years, with a range of 22–60 years. Of these physicians, 760 (49.8%) were males and 767 (50.2%) were females; 408 (26.7%) were single, 1,060 (69.4%) were married, 51 (3.4%) were divorced, and 8 (0.5%) were widowed. The number of participants with junior high school education, senior high school education, undergraduate education, and postgraduate education as the highest academic qualification were 12 (0.8%), 23 (1.5%), 318 (20.8%), and 1,174 (76.9%), respectively. Moreover, there were 444 (29.1%), 372 (24.4%), 197 (12.9%), 167 (10.9%), 226 (14.8%), and 121 (7.9%) participants recruited from the internal medicine department, surgical department, gynecology and pediatrics department, ophthalmology and otorhinolaryngology department, and medical technology department as well as others, respectively. Of these participants, 230 (15.1%) and 131 (8.6%), respectively, had attained a lower 3,000 Yuan income and an over 10,000 Yuan income each month, and 485 (31.8%), 430 (28.2%), and 251 (16.3%) had attained an income of 3,000–5,000 Yuan, 5,000–7,000 Yuan, and 7,000–1,000 Yuan, respectively. Ethics approval to undertake this study was granted by the Research Ethics Committee of Harbin Medical University. We obtained consent from each hospital involved in the research processes. All participants gave informed consent to the researchers before the survey, and participants’ personal information was kept confidential.

### Measurements

Transformational leadership questionnaire (Wang et al., 2018), created by Chaoping Li in Chinese context and based on Multifactor Leadership Questionnaire, is used to assess the transformational leadership level of physicians’ department leader. There are four dimensions (moral modeling, charisma, articulate vision, and individualized consideration) and 26 items. All items are evaluated from score 1 (strongly disagree) to score 5 (strongly agree). A higher score indicates a higher level of transformational leadership. The questionnaire has good reliability and validity, and the Cronbach’s α coefficient is 0.982 in this study.

Physicians’ performance is measured using the 33-item Chinese Employee Job Performance Questionnaire, which is based on the questionnaires designed by Motowidlo and Van Scotter (1994). These 33 items are evaluated from score 1 (never) to score 5 (all the time) to assess followers’ task performance, interpersonal facilitation, job dedication, etc. A higher score indicates a higher level of job performance. In the present study, Cronbach’s α coefficient for the questionnaire is 0.957, indicating that the 33-item Chinese Employee Job Performance Questionnaire is applicable to this study.

The Achievement Motives Scale (Man et al., 1994), designed by Roald Nygard and Torgrim Gjesme and developed by Renmin Ye, is used in this study to measure the achievement motivation of Chinese physicians. There are 30 items, which are evaluated from score 1 (strongly disagree) to score 4 (strongly agree). It includes two dimensions: the motivation to pursue achievement (MS) and the motivation to avoid failure (MF). A higher score indicates a higher level of MS and MF. In the present study, Cronbach’s α coefficients for the MS and MF are 0.894 and 0.924, respectively.

The coping styles of physicians are measured with the Trait Coping Style Questionnaire (Zhou et al., 2016), which is designed by Qianjin Jiang. There are 20 items evaluated from score 1 (strongly disagree) to score 5 (strongly agree). It includes two dimensions: PC and NC. A higher score indicates a higher level of PC and NC. In the present study, Cronbach’s α coefficients for the PC and NC are 0.828 and 0.887, respectively.

### Data Analysis

All data analyses were performed by SPSS 24.0 and Mplus 7.0. The results were evaluated at a significance level of *p* < 0.05 (two-tailed).

Descriptive statistics were used to describe the demographic characteristics (sex, age, marital status, education, department category, and income) of participants. Pearson’s correlation was performed to identify the associations between transformational leadership, MS, MF, PC, NC, and job performance.

A hierarchical linear model was used to test the positive effect of transformational leadership on physicians’ performance. Furthermore, a cross-level mediation model was conducted to explore the multiple mediation effects of achievement motivations (MS and MF) and coping styles (PC and NC). Specifically, in this study, we first tested the hypothetical model to discover the significant and non-significant paths; then, we re-estimated the significant paths and modified the model to determine the final cross-level mediation mechanisms of the link between transformational leadership and physicians’ performance.

## Results

### Descriptive Statistics and Pearson’s Correlation Analyses

Table 1 presented the means, standard deviation, and correlations between study variables (transformational leadership, MS, MF, PC, NC, and job performance). The score of transformational leadership was 101.56 ± 6.42 (ranging from 74.67 to 124.00), with a total score of 130, indicating that transformational leadership was applied well in Chinese hospital settings. Moreover, physicians’ performance was significantly associated with transformational leadership, MS, MF, PC, and NC (*p* < 0.05). Besides, transformational leadership, MS, MF, NC, and PC were significantly related to each other (*p* < 0.05), except for the relationships between MS and NC, MF, and PC (*p* > 0.05).

**TABLE 1:** Means, standard deviations, and correlations for variables.

	M	SD	1.	2.	3.	4.	5.	6.
1. TL	101.56	6.42	1					
2. MS	40.01	7.13	0.065*	1				
3. MF	38.42	7.49	−0.125**	0.158**	1			
4. PC	35.74	5.38	0.141**	0.332**	–0.026	1		
5. NC	30.80	7.24	−0.154**	0.033	0.417**	0.135**	1	
6. Physicians’ performance	127.49	16.36	0.191**	0.473**	−0.104**	0.492**	−0.184**	1

*p < 0.05, **p < 0.01 (two-tailed).

### A Hierarchical Linear Model for the Effect of Transformational Leadership

The present study developed an HLM model in which transformational leadership as a team-level predictor affected physicians’ performance after controlling demographic variables (including sex, age, marital status, education, and income in the individual level and department category in the team level). As Table 2 showed, transformational leadership had a cross-level positive effect on physicians’ performance in Chinese hospitals (β = 1.524, *p* < 0.05).

**TABLE 2:** Hierarchical linear model for the effect of transformational leadership on physicians’ performance.

Variables	Estimate	S.E.	*P* value
**Individual level**			
Sex	0.377	0.993	0.704
Age	0.359	0.065	0.000
Marital status	−1.049	0.634	0.098
Education	1.349	1.100	0.220
Income	0.161	0.474	0.734
**Team level**			
Department category	−0.416	0.285	0.145
TL	1.524	0.274	0.000

### Cross-Level Mediations of Achievement Motivations and Coping Styles

**FIGURE 1:**
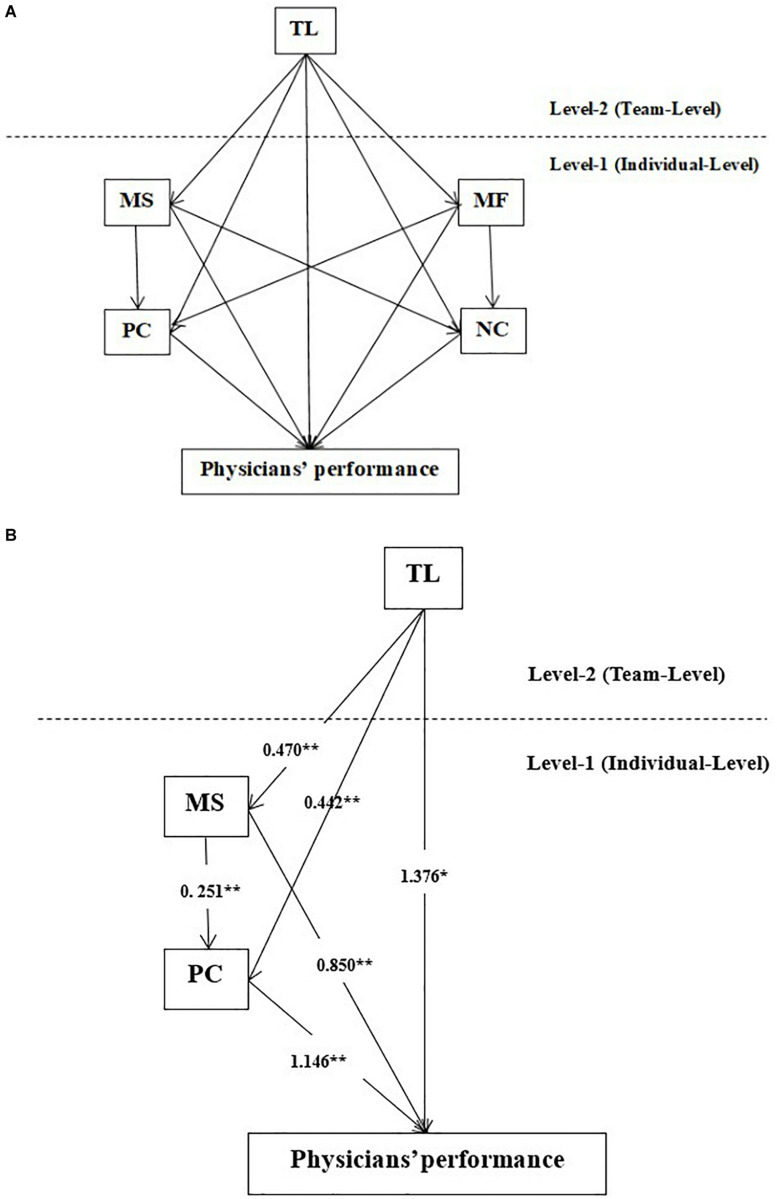
Hypothetical model **(A)** and final model **(B)**. *p < 0.05, **p < 0.01 (two-tailed).

This study performed a cross-level mediation model to test the hypothetical model, and results were presented in Table 3. After removing non-significant paths, we re-estimated the significant paths and modified the model to determine the final cross-level mediation mechanisms of the association between transformational leadership and physicians’ performance. As Figure 1B showed, the positive effect of transformational leadership on physicians’ performance was mediated by MS and PC (*p* < 0.05). Specifically, there were four paths as following: ➀ Transformational leadership → Physicians’ performance, ➁ Transformational leadership → MS → Physicians’ performance, ➂ Transformational leadership → PC → Physicians’ performance, and ➃ Transformational leadership → MS → PC → Physicians’ performance. Additionally, according to path coefficients of the final model present in Figure 1B, the path “Transformational leadership → PC → Physicians’ performance” had the strongest indirect effect (0.442 × 1.146 = 0.507).

**TABLE 3:** Cross-level mediations of achievement motivations and coping styles.

Paths	Estimate	S.E.	*P* value
**Individual level**			
MS → Physicians’ performance	0.860	0.058	0.000
MF → Physicians’ performance	−0.075	0.062	0.229
PC → Physicians’ performance	1.136	0.072	0.000
NC → Physicians’ performance	−0.415	0.053	0.000
MS → PC	0.250	0.021	0.000
MS → NC	−0.061	0.027	0.205
MF → PC	−0.043	0.022	0.047
MF → NC	0.368	0.027	0.000
**Team level**			
TL → Physicians’ performance	1.461	0.254	0.000
TL → MS	0.504	0.192	0.009
TL → MF	−0.314	0.175	0.072
TL → PC	0.478	0.104	0.000
TL → NC	−0.284	0.160	0.076

Moreover, the statistical goodness of fit of model was as follows. In the hypothetical model, values of chi-square, df, CFI, TLI, RMSEA, and SRMR were 139.250, 27, 0.931, 0.795, 0.052, and 0.027, respectively. In the HLM model, values of chi-square, df, CFI, TLI, RMSEA, and SRMR were 29.017, 9, 0.961, 0.914, 0.038, and 0.000, respectively. In final model, values of chi-square, df, CFI, TLI, RMSEA, and SRMR were 78.021, 43, 0.979, 0.965, 0.023, and 0.032, respectively.

## Discussion

Transformational leadership plays a crucial role in health care settings, particularly during major public health emergencies. The present study indicated that transformational leadership was applied well in Chinese hospitals and discovered the cross-level underlying mediation mechanisms of the positive impact of transformational leadership on physicians’ performance. First of all, consistent with other researches (Gabel, 2012; Lievens and Vlerick, 2014), transformational leadership had a cross-level direct positive effect on physicians’ performance. Most importantly, extending previous work, this study found differential processes in how physicians changed their motivations and coping styles under transformational leadership, which eventually led to differences in their job performance. Because there were limited empirical data in Chinese physicians from past studies, our findings provided a better understanding of this relationship by exploring cross-level mediation processes *via* physicians’ achievement motivations and coping styles.

### The Direct Effect of Transformational Leadership on Physicians’ Performance

This study demonstrated that transformational leadership had a significantly cross-level positive impact on physicians’ performance, corroborating previous research that documented that transformational leadership was a key factor to improve employees’ performance (Miao and Cao, 2019). The importance of transformational leadership in medical settings is well recognized, and it always creates favorable outcomes for physicians, nurses, hospitals, etc. (Brewer et al., 2016; Boamah, 2018). The health care system is unique, complex, and politically sensitive because of its impacts on people’s health and well-being. In particular in China, hospitals face fresh challenges in delivering affordable and high-quality care to meet growing health needs (Nong and Yao, 2019). The Chinese government has announced a series of health care system reform to improve public health services, establish essential medicines program, and reform public hospitals (Yip and Hsiao, 2014). Moreover, hospitals adapt to meet the needs of evolving health care practice when faced with major public health challenges, such as outbreaks of influenza-like illness, severe pneumonia, etc. (Sun et al., 2018). As the main medical service providers, Chinese physicians have to work for a 1.4 billion population and adapt to a series of public health challenges in their work; they may suffer from burnout by overwork (Wen et al., 2016). The rapid changes in Chinese society require a highly skilled and resilient health service management workforce. Transformational leadership is necessary to improve the quality of health care with greater involvement and adaptability. The application of transformational leadership is appropriate to physicians in different departments. Transformational leaders can effectively deal with a complex and rapidly changing working environment in health care settings. The leaders act as role models for physicians to show an idealized influence of transformational leadership. They express an organization’s vision, stimulate emotion, and provide support for their subordinates (Wu and Lee, 2020). Transformational leadership contributes to the improvement of physicians’ job performance and quality of health care services. More attention is warranted toward medical leadership, particularly transformational leadership, which has been empirically proven to foster personal development and performance.

### The Cross-Level Mediation Effects of Achievement Motivations and Coping Styles

In the cross-level study design, the results of this study indicated that transformational leadership not only had a direct effect on physicians’ performance but also had indirect effects *via* achievement motivations and coping styles. There were three crucial paths through which the cross-level mediation model of transformational leadership on Chinese physicians’ performance: ➀ Transformational leadership → MS → Physicians’ performance, ➁ Transformational leadership → PC → Physicians’ performance, and ➁ Transformational leadership → MS → PC → Physicians’ performance.

In this study, separate mediations of MS and PC were statistically significant in the relationship between transformational leadership and physicians’ performance. In health care settings, managers’ leadership style is represented by a set of attitudes, behaviors, beliefs, and values, which has a profound impact on health workers’ cognition and behavior patterns (Jodar et al., 2016). Transformational leaders gave every physician identification and stimulated them to share an organization vision as inspirational motivation. By creating progressive relationships, transformational leaders improve the self-stimulated achievement motivation of their subordinates in order to motivate them to achieve work well-being and perform in a better way (Miao and Cao, 2019; Stuber et al., 2019). A physician with high MS aspired to pursue success, enjoyed their works, and was willing to take some risks to achieve his or her achievement goals. In the health care system, transformational leadership promotes a better practicing environment for workers to be professional and enhance their performance. On the other hand, effective leadership improves not only physicians’ work motivation but also their work coping styles. In health care settings, transformational leaders encourage their subordinates to view problems from a new perspective and work hard (Lin et al., 2019). Physicians made positive self-regulation and tried to translate their own NC strategies into PC strategies when they encountered difficulties in the work setting. Transformational leaders’ behavior is essential to promote subordinates’ active behavior and personal growth (Lin et al., 2020). Under transformational leadership, physicians would positively respond to the tasks and problems, trying their best to overcome difficulties in medical activities. As a result, job performance could be significantly improved.

Furthermore, the serial-multiple mediations of achievement motivations and coping styles were tested in this study and proved to be statistically significant in the positive impact of transformational leadership on physicians’ performance. Specifically, the results of this study showed that transformational leadership was sequentially associated with increased MS first and then increased PC, and finally contributed to increased physicians’ performance. In the Chinese health care system, the heavy workload and fast-paced working conditions, such as long work hours and high work requirements, have brought a lot of work pressure to employees (Lu et al., 2018). Given the importance of patient safety, physicians are required more professional knowledge and skills, and responsibility. Transformational leaders improve subordinates’ work performance by idealized influence, inspirational motivation, inspirational motivation, and individualized consideration (Asif et al., 2019). As discussed before, transformational leadership was beneficial to physicians’ problem solving and cognitive restructuring strategies. Transformational leaders motivate physicians’ achievement motivation and promote them to pursue more outstanding achievements, contributions, honor, social status, etc. Physicians, who had a higher level of MS, devoted more energy to work and career and demanded a high standard to pursue high-quality medical service (Tsounis et al., 2014; Völker and Hunchangsith, 2018). With high-level achievement motivation, physicians changed their work coping styles energetically. They positively responded to the tasks and problems at work and chose a suitable treatment plan based on balancing advantages and disadvantages, achieving maximum success within the medical rule allowed. Unexpectedly, our results did not support the significant cross-level effect of transformational leadership on physicians’ performance by affecting MF and NC in a proper sequence. This might indicate that in Chinese health care settings, transformational leadership played a significant role in improving positive psychological quality, but it was ineffective in reducing negative psychological response strategies.

Additionally, the path “Transformational leadership → PC → physicians’ performance” had the strongest indirect effect of transformational leadership on physicians’ performance. Among Chinese physicians, it might be better to change coping styles than their motivations when medical leaders tried to improve physicians’ performance. In general, a transformational leader could effectively improve physicians’ PC strategies by setting an example and sharing working methods, which should be highly valued.

### Implications

Public health challenges have required medical leaders to lead effectively toward the new setting. As an essential contribution to the practice of human resource management, the current study provided valuable insights into the impact of transformational leadership on physicians’ performance and first reported a cross-level underlying mediation mechanism of this relationship with achievement motivations and coping styles among Chinese physicians. Our findings suggested that transformational leadership should be more utilized in health care management. Medical leaders could receive systematic transformational leadership training when necessary. Creating a good working environment, inspiring achievement motivation, and enhancing PC strategies in physicians, transformational leaders made every effort to improve the quality of health care. Our model gave advice to public health managers, policymakers, and other health professionals in discussion around public health leadership. Overall, it is of great social significance to apply transformational leadership in health care settings, which benefits physicians’ performance, quality of medical service, and patient safety.

### Limitations

Although this study contributed to future research and public health management practice, some limitations needed to be noticed. First, the sample source was a limitation of this study, which collected data from hospitals in Harbin. The current research was a single-country study, and the impact of transformational leadership on physicians’ performance should be examined based on cross-national surveys in the future. Second, since the study is cross-sectional, we cannot draw real dynamic modeling and conclude that the associations between factors are truly causative. Moreover, given the self-report instruments, there might be response bias. Improvement of assessment instruments is necessary for future studies.

## Conclusion

In health care settings, transformational leadership could effectively enhance physicians’ performance. The current study suggested that transformational leadership should be more applied in public health settings, and leaders should enhance physicians’ performance by promoting individual development, especially achievement motivation and PC style.

## Data Availability Statement

The original contributions presented in the study are included in the article/Supplementary Material, further inquiries can be directed to the corresponding author/s.

## Ethics Statement

The studies involving human participants were reviewed and approved by the Research Ethics Committee of Harbin Medical University. The patients/participants provided their written informed consent to participate in this study.

## Author Contributions

YY and HC: conceptualization. HC: methodology, software, formal analysis, writing–original draft preparation, and visualization. HC, BQ, JZ, DC, and YY: validation. HC, JZ, XY, XQ, ZQ, XS, and EZ: investigation. YY: resources, data curation, supervision, and project administration. HC, BQ, DC, and YY: writing–review and editing. All authors have approved the submitted version and agreed to be personally accountable for the author’s own contributions.

## Conflict of Interest

The authors declare that the research was conducted in the absence of any commercial or financial relationships that could be construed as a potential conflict of interest.

## Abbreviations

TL: transformational leadership
MS: motivation to pursue achievement
MF: motivation to avoid failure
PC: positive coping
NC: negative coping.
